# Is conflict adaptation an illusion?

**DOI:** 10.3389/fpsyg.2015.00172

**Published:** 2015-02-18

**Authors:** James R. Schmidt, Wim Notebaert, Eva Van Den Bussche

**Affiliations:** ^1^Department of Experimental Clinical and Health Psychology, Ghent UniversityGhent, Belgium; ^2^Department of Experimental Psychology, Ghent UniversityGhent, Belgium; ^3^Department of Psychology, Vrije Universiteit BrusselBrussels, Belgium

**Keywords:** conflict adaptation, contingency learning, cognitive control, attention, timing, expectancies, proportion congruent, congruency sequence effect

Conflict adaptation theory is one of the most popular theories in cognitive psychology. The theory argues that participants strategically modulate attention away from distracting stimulus features in response to conflict. This idea was particularly popularized with the publication of the conflict monitoring model of Botvinick et al. (2001). Although the conflict adaptation view is able to explain a wide range of results with a seemingly intuitive set of mechanisms, some researchers have expressed skepticism. The paradigms used in the study of conflict adaptation typically require the manipulation of stimulus frequencies, sequential dependencies, time-on-task regularities, and various other task regularities that introduce the potential for learning of conflict-unrelated information (for a review, see Schmidt, 2013a). This raises the possibility that although the *data patterns* (e.g., reduced congruency effects following incongruent trials) might be very real, the conflict adaptation *mechanism* typically used to explain them might be an illusion.

This research topic produced 17 articles from 39 authors. The contributions span a range of tasks, broadly divided into work on the congruency sequence effect (CSE) and various versions of the proportion congruency (PC) task. Duthoo et al. (2014) provide an updated review of the CSE literature, including considerations regarding difficulties with learning confounds that will need to be overcome in future research. Braem et al. (2014) provide a review and synthesis of work on cross-task CSEs, and they highlight a potentially important role of similarity in task context. Egner (2014) provides another review wherein it is argued that “learning biases” and conflict adaptation may be two expressions of a similar learning mechanism, the latter merely more abstract than the former.

The role that feature bindings play in confounding the CSE has been a central issue since seminal papers by Mayr et al. (2003) and Hommel et al. (2004). Spapé and Hommel (2014) further this work with a paradigm in which target location boxes rotate to new positions on the screen between trials, with results seeming to indicate a dependency of CSEs on bindings between stimuli. Van Lierde et al. (2014) present masked-priming experiments that produced an irregular CSE pattern when feature repetitions were included, but a regular CSE in the error rates with feature repetitions excluded. Wendt et al. (2014) present data to suggest that controls for feature bindings may be insufficient in cross-task CSEs when there is a semantic overlap between features in the two sub-tasks.

As early as the very first observation of a CSE, the role of expectancies about a repetition vs. alternation of congruency type (i.e., congruent vs. incongruent) has been discussed (Gratton et al., 1992). Jiménez and Méndez (2014) present evidence to suggest that conscious expectancies only influence behavior when participants are explicitly probed for their expectancies. In a less traditional paradigm using alphabet verification and serial reaction tasks, Gaschler et al. (2014) present evidence for the transfer of control demands from one learning task to another.

Some key articles have illustrated the major issues with contingent regularities in PC and CSE tasks (e.g., Schmidt and Besner, 2008; Schmidt and De Houwer, 2011; Mordkoff, 2012). Hazeltine and Mordkoff (2014) observe that robust effects of contingencies fully account for item-specific PC (ISPC) effects (see also, Schmidt, 2013b). They further observe sequential modulations of both contingencies and congruency on the CSE. In contrast, Blais et al. (2014) suggest that contingency biases and “congruency switch” biases are unlikely to contribute to the CSE, though Schmidt (2014b) contests the interpretation of the data in a response paper.

A particularly interesting, howbeit controversial, development in the PC literature came with the suggestion that adaptation to conflict might occur in an item-specific (Jacoby et al., 2003) or context-specific fashion (Corballis and Gratton, 2003; Crump et al., 2006). Schmidt et al. (2014) present a non-conflict analog to the context-specific PC effect and argue that the “context-specific proportion easy” effect they observe is consistent with the notion that context-specific rhythms might explain context-specific PC effects. Atalay and Misirlisoy (2014) investigate the ISPC effect with different asynchronies (SOA) between targets and distracters. Generally consistent with a contingency learning perspective, they observe robust ISPC effects across lags, except when the distracting word came too late after the color.

Entel et al. (2014) investigate the influence of explicitly instructed contingencies on PC effects. They suggest that instructions alone might trigger proactive control, while also arguing an important role for contingencies. Hasegawa and Takahashi (2014) investigate block-wide PC effects and CSEs in a masked priming paradigm. They observed block-wide PC effects even with minimal stimulus awareness, but evidence for CSEs was limited to errors.

The topic closes with two opinion articles. Schmidt (2014a) discusses yet another potential caveat with contingency biases in cognitive control paradigms: if some stimuli are highly predictive of a response, whereas others are not, then differences in stimulus informativeness can lead to attentional capture biases. Finally, Levin and Tzelgov (2014) discuss an interesting distinction between task and informational conflict, and how this distinction might have important implications for theorizing in the cognitive control literature.

The range of perspectives presented in this research topic are as diverse as the questions assessed. Regarding the main question of interest (i.e., “Is Conflict Adaptation an Illusion?”), some authors argue that the answer is a resounding “yes,” others argue that evidence for conflict adaptation is clear, and yet others fall somewhere in between. Whether or not conflict adaptation is merely an illusion is still an open question, but the contributions of the current research topic add interesting new layers to the debate. We hope that this research topic will open new avenues for research in the area that may lead to more definitive answers.

## Conflict of interest statement

The authors declare that the research was conducted in the absence of any commercial or financial relationships that could be construed as a potential conflict of interest.
